# Pure Food in Texas

**Published:** 1906-08

**Authors:** 


Pure Food in Texas.—*Holland's Magazine,* Dallas, Texas, has come to the aid of the medical profession in a crusade against food adulteration and is doing good work. Some startling facts are published in *Holland's* for August. In September *Holland's* will take up the subject of milk. The people of Texas, or at least those in the larger cities and towns, are being forced to buy a very large amount of milk which is treated with formaldehyde and other preservatives. Milk adulteration is one of the greatest crimes which can be committed against the public since the life and health of thousands of helpless children in the larger cities and towns are absolutely dependent upon the milk supply furnished by the dairymen.

*Holland's* has had a chemist at work for some time making analyses of milk and other foods purchased in the open market in various cities and towns and a detailed report of his findings in regard to milk, it is stated, will be given in *Holland's* for September. The percentage of preserved or "embalmed" milk sold is said to be appalling.

The investigations being made by *Holland's* are not confined to milk, but embrace a large number of food necessities, many of which are said to be grossly adulterated, and the samples being examined are not from Texas alone, but also from other Southern and Southwestern States.

The national government recently enacted a pure food bill, but this does not affect local sales in the various States, and *Holland's* is to be commended for the stand it has taken in favor of the enforcement of pure food laws in States and municipalities.

*Bad Milk in Texas.*—Regarding the sale of impure milk in Texas, *Holland's Magazine* says editorially:

"In the adoption of the pure food and meat inspection laws the national government has taken a long step toward assuring the public of a clean food supply, but no matter how drastic the national laws, there must be stringent State and municipal regulation as well if the people are to be given adequate protection. It is not the province of the national government to control local sales or to interfere with local police regulations. The States, cities and towns must adopt laws themselves for the protection of their own people.

"Many towns and cities and some States are wholly without pure food laws or else such laws are inoperative for the reason that no provision or appropriations are made for their enforcement. Texas is one of the States in which milk, meats and other food products are sold without inspection, save such as may be imposed by different municipalities. To find just what the people of Texas were eating and drinking, *Holland's* recently sent to a number of the larger cities and towns one of its representatives accompanied by an expert analytical chemist. Samples of various food products were purchased in the open market and analyses are being made by the chemist. While these analyses have not yet been completed, the work has so far advanced as to justify the assertion that the wholesale adulteration of some food products is common; the extent to which milk containing formaldehyde or other harmful preservatives is sold is not only so great as to be alarming, but the percentage of impure milk in some Texas cities is so high as to be almost beyond belief.

"In the September number of *Holland's* the results of the chemist's analyses of milk samples bought in different cities will be given in full and impurities found in other foods will be discussed in later issues."

---

The following resolution was adopted by the North Texas Medical Association at its recent meeting in Denison, Texas:

WHEREAS, One of the most serious obstacles encountered by physicians in regular practice is the substitution of drugs not of standard purity and strength for those named in prescriptions, and,

WHEREAS, Such substitutions are a menace not only to the reputation and practice of the physician, but are a potent source of danger to the public health, and,

WHEREAS, It has come to our knowledge that *Farm and Ranch* and *Holland's Magazine,* both published by the Texas Farm and Ranch Pub. Co., of Dallas, Texas, are engaged in gathering data and information on which to base a strenuous campaign in favor of the sale of pure drugs and against the drug substitution evil to the end that the public health may be better protected, it is

*Resolved,* That the North Texas Medical Association fully indorses the motives of such campaign and that its members pledge their cordial and hearty co-operation and support in their respective communities to *Farm and Ranch* and *Holland's Magazine* in such action as may be taken looking to the elimination of the substitution evil and the preservation of the health of the public.

